# Solubility-improved and antitumor activity of (-)-cannabidiol conjugates

**DOI:** 10.1186/s42238-026-00453-5

**Published:** 2026-05-28

**Authors:** Daniel Crespo, Javier Moreno, Cristina Mesas, Amelia Díaz, Iván Cheng-Sánchez, Francisco Sarabia, J. Manuel López-Romero, Consolación Melguizo, José Prados

**Affiliations:** 1 Dpto. de Química Orgánica, Facultad de Ciencias, Universdad de Málaga, Boulevard Louis Pasteur 31, Málaga, 29071 Spain; 2 Instituto Universitario de Materiales y Nanotecnología, IMANA, Universidad de Málaga, Campus de Teatinos, Málaga, 29071 Spain; 3 Instituto de Investigación Biosanitaria de Granada (ibs. GRANADA), Granada, 18014 Spain; 4 Institute of Biopathology and Regenerative Medicine (IBIMER), Center of Biomedical Research (CIBM), University of Granada, Granada, 18100 Spain; 5 Dep. of Anatomy and Embryology, Faculty of Medicine, University of Granada, Granada, 18071 Spain

**Keywords:** CBD, Chemical conjugates, Resistant cancer, Cytotoxicity, Solubility

## Abstract

**Background:**

(-)-Cannabidiol (CBD) is a naturally occurring terpenoid belonging to the cannabinoid family, which is isolated from the *Cannabis sativa* L. plant. It possesses significant therapeutic potential, providing minimal side effects and no psychoactive activity. However, CBD applications are limited by a poor aqueous solubility and low bioavailability.

**Objective:**

To address these limitations and investigate the impact of structural modification on solubility, we plan in this work the synthesis of a series of CBD conjugates along with the evaluation of their antitumor activity.

**Methods:**

Conjugates are characterized by Nuclear Magnetic Resonance (NMR) and Mass Spectrometry (MS) techniques, along with the solubility in water, glycerine, *n*-hexane and isooctane evaluated by High-Performance Liquid Chromatography (HPLC). The antitumoral activity of the CBD conjugates has been tested using cytotoxicity (IC_50_), cell migration and cell colony formation assays against lung adenocarcinoma cells A549.

**Results:**

Specifically, *oligo*(ethylene glycol)-, alkyl- and *L*-valine-functionalized CBD derivatives are synthesized *via* selective esterification of its phenolic groups in good yields. Solubility profiles revealed a marked improvement compared to CBD. Notably, *oligo*(ethylene glycol) derivatives significantly enhanced solubility in water and glycerine, with 1b exhibiting a 14-fold increase in water solubility. The *L*-valine bis-conjugated derivative 4 also exhibited substantially improved solubility across all tested solvents, reaching up to a 13-fold increase in glycerine. In contrast, alkyl conjugates 2a,b showed only modest improvements. In addition, the in vitro evaluation against A549 revealed improved cytotoxic activity for 1a, 1b and 4 compared to native CBD, and with 1a additionally displaying antimigratory and colony formation inhibitory effects.

**Conclusions:**

Collectively, our results highlight the critical role of conjugate structure in modulating physicochemical and biological properties of CBD and underscore the potential of these CBD conjugates as promising candidates for further pharmacological investigation and development.

**Supplementary Information:**

The online version contains supplementary material available at 10.1186/s42238-026-00453-5.

## Background

(-)-Cannabidiol (CBD) is a natural compound belonging to the cannabinoid family, products isolated from the *Cannabis sativa* L. plant. Among the various compounds identified from this plant, approximately 66 have been identified as cannabinoids, the most abundant and important are tetrahydrocannabinol (Δ^9^-THC), cannabigerol (CBG), cannabinol (CBN), cannabichromene (CBC) and CBD (Huang et al., 2023; Singh et al. 2025).

CBD exhibits numerous therapeutic activities, including anti-inflammatory, antibiotic, analgesic, antitumor, and anxiolytic properties. Furthermore, this cannabinoid is well-tolerated by patients and has minimal side effects across a wide range of doses (O’Sullivan et al., 2023). Unlike other widely studied phytocannabinoids, such as Δ^9^-THC, CBD does not have psychoactive effects, a fact that, combined with its biological activity, has generated increasing interest in its research (Odieka et al. 2022; Nelson et al. 2020; Mesas et al. 2025). Moreover, in light of the numerous benefits discovered about CBD, many countries have legalized its use for medicinal purposes (Bobitt et al. 2025). In fact, it is currently used as part of treatments for neurodegenerative diseases such as epilepsy, Parkinson’s, and Alzheimer’s (von Wrede et al. 2021). Its use as an additive in foods and beverages is also becoming increasingly common, and it can be found from infusions and soft drinks to baked goods, chocolates, and gummies (Christinat et al. 2020). Regarding cancer therapies, it has shown potential not only for alleviating symptoms such as weight loss, vomiting, and pain, but also for promoting antitumor effects, such as inhibiting cell proliferation, inducing apoptosis, and altering tumour neovascularization (Kovalchuk et al., 2020).

The biological activities of CBD are primarily due to its interaction with the endocannabinoid system. This neurotransmission system present in humans, participates in the development of the central nervous system and plays an important role in regulating multiple cognitive and physiological processes. It is composed mainly of cannabinoid receptors, CB1 and CB2, endogenous ligands known as endocannabinoids, and proteins that transport, synthesize, and degrade these ligands (Hui-Chen et al., 2021). Similarly, it should be noted that these receptors regulate various biological functions such as appetite and metabolism, which can be very useful when patients undergo treatments with associated symptoms such as chemotherapy (Heider et al. 2022). On the other hand, CB2 receptors are particularly related to the immune system, being present in organs such as the spleen, thymus, spinal cord, and various peripheral tissues (Bobitt et al. 2025). Specifically, these receptors are involved in the control and regulation of cytokinesis in immune cells, which can help prevent the development of cancer by preventing uncontrolled cell proliferation (Heider et al. 2022). Furthermore, CBD also binds with affinity to several receptors, notably the transient receptor potential vanilloid (TRPV), peroxisome proliferator-activated receptor gamma (PPARγ), G protein-coupled receptor (GPR55), and serotonin receptor (5-HT1A). CBD exhibits allosteric binding activity with these receptors, binding as an inverse agonist or antagonist to the CB1 receptor, acting as an antagonist at CB2 and GPR55 receptors, and behaving as a partial agonist at TRPV and 5-HT1A receptors (Bobitt et al. 2025). Finally, the antipsychotic properties of CBD are related to the inhibition of fatty acid amide hydrolase and its interaction with 5-HT1A and TRPV1 receptors, while its antidepressant and anxiolytic activity are attributed to its interaction with the 5-HT1A receptor (Bobitt et al. 2025). Chemically, it should also be noted that CBD exhibits antioxidant properties due to the presence of two hydroxyl groups in its structure.

One of the potential applications of CBD is the treatment against lung cancer, the deadliest form of cancer with 1.8 million deaths in 2022 (Guo et al. 2024). Nowadays, chemotherapy is the principal treatment in late-stage diagnosed patients, and is based on the use of cytotoxic compounds or drugs including antimetabolites, taxanes or platinum-based compounds (Lemjabbar-Alaoui et al., 2015), and most of the actual treatments for late-stage lung cancer are not effective enough, leading the study and development of alternative molecules and personalized therapy to reduce the secondary effects of the traditional chemotherapy and decrease the mortality (Wang et al. 2021). CBD have some useful secondary effects in the cancer treatment as anti-nausea and analgesic but also can interact directly with cancer cells due to the over-expression of the endocannabinoid system molecules TRPV1, CB1 or CB2 in some cancer cell lines, leading to an oxidative stress, antiproliferative and cytotoxic effect (O’Brien et al., 2022). The antitumoral effect of CBD have already been studied in lung cancer cell models in vivo, reducing the tumour growth rate and the CD44 cell mark expression in subcutaneous induced NCI-H1437 cell tumour in mice (Guo et al. 2024). CBD have been tested in clinical trials, but only its analgesic and anti-inflammatory effect, not having clinical evidence of its antitumoral effect (Cásedas et al. 2024).

Such is the pharmaceutical interest in this compound that various research groups have made significant efforts to define new synthetic strategies for a rapid access to the product, avoiding absolute dependence on its natural source.

**Scheme 1:**
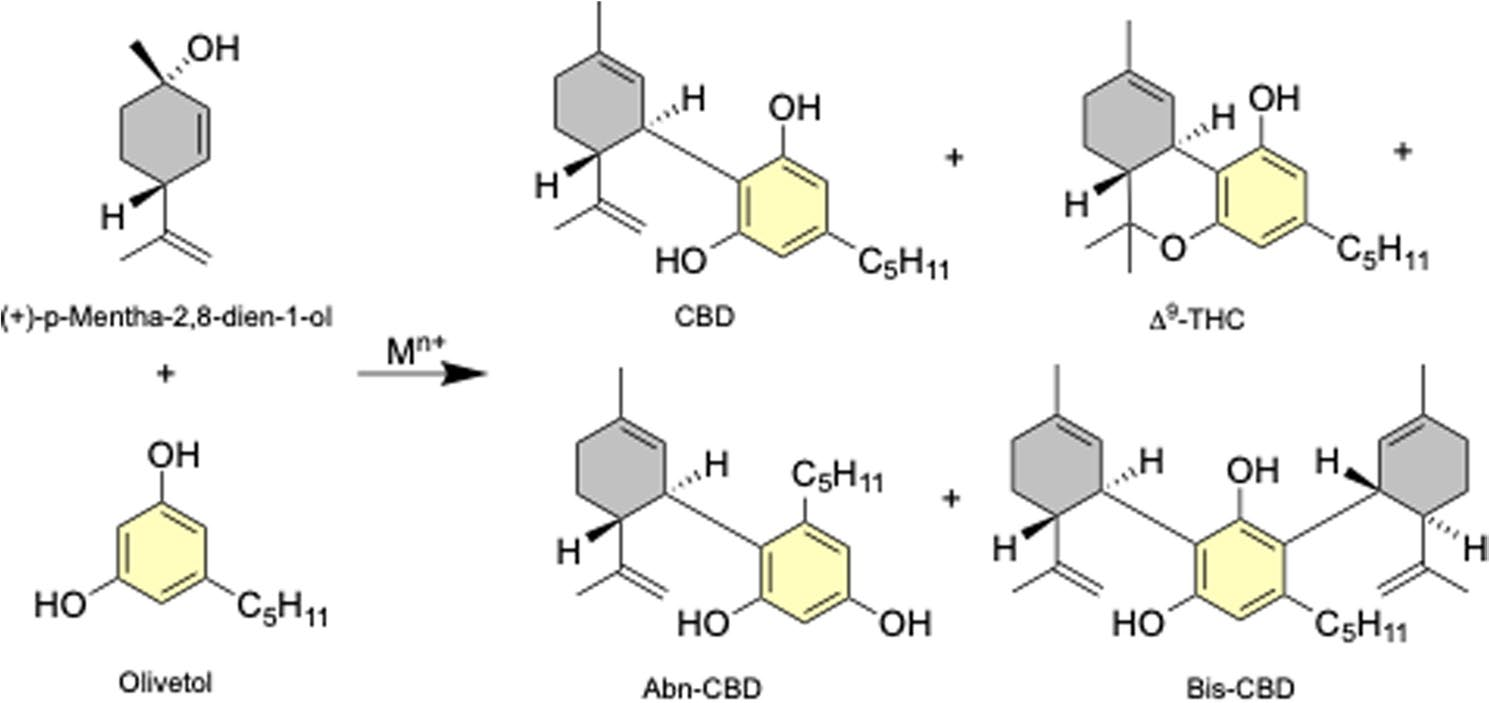
Synthetic strategy for CBD *via* aryl coupling of olivetol and (+)-p-mentha-2,8-dien-1-ol

One of the main synthetic strategies for the industrial production of CBD involves a Friedel-Crafts reaction, in which olivetol is combined with a terpene unit, typically (+)-*p*-mentha-2,8-dien-1-ol, in the presence of different acid catalysts. To date, all reported studies have employed a wide variety of acids, from Brønsted acids to Lewis’s acids (M^n+^ in Scheme 1), achieving moderate yields and low regioselectivities, affording in addition to CBD, compounds Δ^9^-THC, Abn-CBD and Bis-CBD (Scheme 1) (Ramer et al. 2011). However, satisfactory results in terms of both selectivity and chemical yields were obtained when using FeCl_3_·6H_2_O as an acid catalyst. The synthesis yields mostly CBD (89% yield), with no Abn-CBD formation detected and only traces of Δ9-THC and Bis-CBD observed (Moya-Utrera et al. 2024). In any case, biaryl coupling has not been tested for the preparation of derivatives.

The therapeutic and dietary applications of CBD have significant limitations due to its low solubility and bioavailability, its sensitivity to environmental conditions, and its poor emulsifying properties in water at neutral pH and in hydrophilic solvents (Grifoni et al. 2022). CBD is a highly hydrophobic compound, practically insoluble in aqueous solvents, but with good solubility in organic solvents such as methanol or dichloromethane (Pulido-Bonilla et al. 2023). This low water solubility (12.6 mg/L) and its high lipophilicity make its absorption and entry into the bloodstream inefficient, which greatly hinders its clinical use as a drug due to its low bioavailability.

**Scheme 2:**
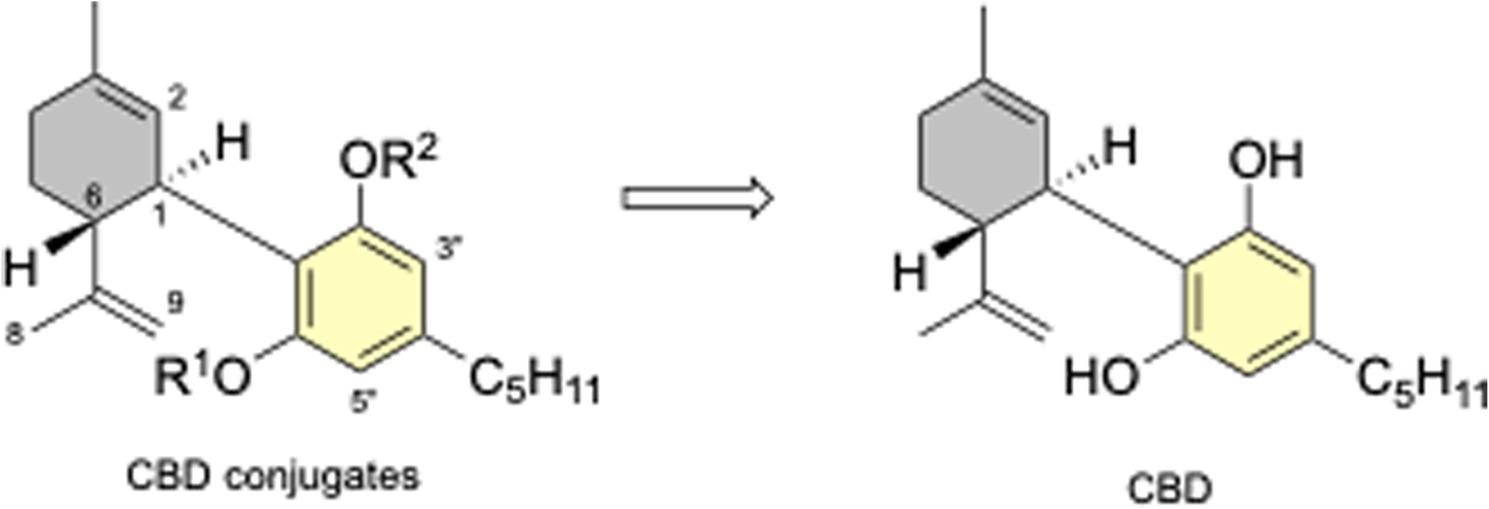
CBD conjugates from CBD

To address this problem, research has been focussed on the synthesis of new water-soluble CBD conjugates and on the development of nanoparticle suspensions and micellar systems (Moya-Utrera et al. 2024; Fuentes-Ríos et al. 2024). The structural modification strategy proposed herein involves the deprotonation of the phenolic hydroxyl groups located on the olivetol moiety under basic conditions, thereby enhancing their nucleophilicity and enabling subsequent nucleophilic substitution with selected electrophilic chains. The “R” substituents (Scheme 2) to be introduced into the CBD scaffold are derived from amino acids, alkyl halides, carbohydrates, and *oligo*(ethylene glycol) (OEG) derivatives. This approach is expected to improve aqueous solubility while preserving the structural integrity of the cannabinoid core and its general activity.

## Methods

### General remarks

Tetrahydrofuran (THF) was distilled from sodium/benzophenone under argon atmosphere, while dichloromethane was distilled over CaH_2_. All other reagents and solvents were purchased from commercial sources and used without further purification. All reactions were carried out under a N_2_ atmosphere using dry, freshly distilled solvents under anhydrous conditions, unless aqueous reagents were used. Reactions were monitored by thin-layer chromatography using 0.25 mm silica gel plates (60 F − 254) using UV light (356 nm) as a visualizing agent, and acidic solutions of cerium and ammonium molybdate or potassium permanganate solutions and heat as developing agents. Column chromatography was carried out using silica gel (60 Å, particle size 230–400 mesh) under compressed air. All solvents were evaporated at reduced pressure by using rotary evaporator. Millipore water was used in all experiments.

Melting points (Mp) were determined with a Gallenkamp instrument. Ultraviolet measurements were recorded on a Biochrom Libra S22 spectrophotometer. HPLC were carried out in an Thermo Scientific Ultimate 3000 equipped with a photodiode array detector. Separation was achieved on an Agilet ODS column (250 mm x 4.6 mm; 5 μm) operating at 25–30 °C. The mobile phase consists of water (phase A) and acetonitrile (phase B) in a gradient of A65:35B. Flow at 1.0 mL/min. Injection volume 10 µL. Conjugates were detected at 280 nm. High-resolution mass spectrometry (HRMS) was performed using a Thermo Scientific Q Exactive Orbitrap detector, with ESI ionization applied at 4 kV in positive polarity. HRMS signals are reported to four decimal places and are within a range of ± 5 ppm within theoretical values. ^1^H and ^13^C NMR spectra were recorded on a Bruker DPX-500 MHz spectrometer and calibrated using a residual non-deuterated solvent as an internal reference. Chemical shifts are reported in ppm, with resonance resulting from incomplete deuteration of the solvent used as an internal standard (CDCl_3_: 7.26 ppm). The following abbreviations are used to explain multiplicities: s= singlet, d= doublet, t= triplet, q= quartet, m= multiplet, or a combination of these, and br= broad. Full NMR spectra are included in Supplementary Information document.

### Synthesis

#### Reaction of CBD with oligo(ethylene glycol)s. Synthesis of compounds 1a and 1b(Scheme 3)

A solution of CBD (200 mg, 0.64 mmol, 1 eq.) in anhydrous CH_2_Cl_2_ (8 mL) was prepared in a 100 mL round-bottom flask under an inert atmosphere. To this solution, 2-[2-(2-methoxyethoxy)ethoxy]acetic acid (0.11 mL, 0.70 mmol, 1.1 eq.), *N*,*N*’-dicyclohexylcarbodiimide (DCC, 144.4 mg, 0.70 mmol, 1.1 eq.) and dimethylamino pyridine (DMAP, 7.8 mg, 0.06 mmol, 0.1 eq.) were added while stirring. The reaction mixture was stirred for 12 h at room temperature. After completion of the reaction, monitored by TLC, the mixture was filtered and washed 3 times with CH_2_Cl_2_. Finally, the filtrate was concentrated under reduced pressure and purified by column chromatography with silica gel (15% Et_2_O in hexane, 45% EtOAc in hexane), obtaining compound 1a as a yellow syrup (203 mg, 67% yield). ^1^H NMR (500 MHz, CDCl_3_) δ ppm: 6.55 (s, 1H, Ar-H), 6.42 (s, 1H, Ar-H), 6.00 (br s, 1H, OH), 5.52 (br s, 1H, H-2), 4.58 (s, 1H, H-9), 4.42 (br s, 1H, H-9´), 4.34 (s, 2 H, CH_2_CO), 3.85–3.77 (m, 2 H), 3.72 (t, *J* = 4.7 Hz, 2 H), 3.63 (t, *J* = 4.7 Hz, 2 H), 3.56 (t, *J* = 4.7 Hz, 2 H), 3.46 (s, 1H), 3.38 (s, 3 H, OCH_3_), 2.50–2.42 (m, 3 H, H, CH_2_Ph), 2.20–2.16 (m, 1H), 2.07 (br m, 1H), 1.82–1.70 (m, 3 H), 1.59–1.53 (m, 5 H), 1.33–1.24 (m, 6 H), 0.87 (t, *J* = 6.9 Hz, 3 H). ^13^C NMR (100 MHz, CDCl_3_) δ ppm: 168.95, 155.83, 148.77, 147.23, 143.08, 140.64, 123.22, 119.04, 114.93, 113.92, 111.53, 72.04, 71.19, 70.80, 70.70, 68.64, 59.16, 45.75, 38.03, 35.48, 31.55, 30.58, 30.29, 27.98, 23.71, 22.61, 20.04, 14.13. HRMS *m/z*: [M + Na]^+^ calculated for: C_28_H_42_O_6_Na 497.2879, found 497.2884.

When the reaction was carried out by using 2-[2-(2-methoxyethoxy)ethoxy]acetic acid (0.22 mL, 1.40 mmol, 2.2 eq.), DCC (288.7 mg, 1.40 mmol, 2.2 eq.) and DMAP (7.8 mg, 0.06 mmol, 0.1 eq.), compound 1b was obtained as a white solid (60% yield, 242 mg). Mp 167–169 °C. ^1^H NMR (500 MHz, CDCl_3_) δ ppm: 6.74 (s, 2 H, Ar-H), 5.17 (s, 1H, H-2), 4.52 (s, 1H, H-9), 4.43 (s, 1H, H-9´), 4.35–4.14 (m, 4 H, 2 x CH_2_CO), 3.84–3.76 (m, 4 H), 3.71 (t, *J* = 4.5 Hz, 4 H), 3.66 (t, *J* = 4.5 Hz, 4 H), 3.55 (t, *J* = 4.5 Hz, 4 H), 3.47–3.44 (m, 1H), 3.37 (s, 6 H), 2.59–2.52 (m, 3 H, H, CH_2_Ph), 2.16–2.09 (m, 1H), 2.03–1.98 (m, 1H), 1.93–1.90 (m, 1H), 1.79–1.54 (m, 9 H), 1.31–1.26 (m, 4 H), 0.87 (t, *J* = 6.9 Hz, 3 H). ^13^C NMR (100 MHz, CDCl_3_) δ ppm: 168.70, 156.90, 149.15,147.82, 142.32 133.15, 126.04, 124.40, 111.19, 72.01, 71.20, 70.80, 70.69, 68.60, 59.15, 49.18, 45.74, 38.59, 35.29, 34.06, 31.50, 30.54, 30.40, 28.79, 25.72, 25.06, 23.52, 22.54, 19.88, 14.10. HRMS *m/z*: [M + Na]^+^ calculated for: C_35_H_54_O_10_Na 657.3615, found 657.3626.

#### Reaction of CBD with alkyl halides. Synthesis of compounds 2a and 2b(Scheme 3)

A solution of CBD (500 mg, 1.59 mmol, 1 eq.) and K_2_CO_3_ (659 mg, 4.77 mmol, 3 eq.) in 60 mL of acetone was prepared in a 100 mL round-bottom flask under an inert atmosphere. To this mixture, 1-bromooctane (0.41 mL, 2.39 mmol, 1.5 eq.) was added and the mixture was refluxed for 12 h. The reaction crude was then cooled to room temperature, and the solvent was removed under vacuum. To the resulting residue, CH_2_Cl_2_ and saturated brine were then added. The aqueous layer was extracted with CH_2_Cl_2_ (3 × 10 mL). The organic extract was washed with brine (3 × 10 mL), dried over anhydrous MgSO_4_ and concentrated under vacuum. Finally, the obtained product was purified by column chromatography with silica gel (100% hexane, 1% EtOAc in hexane), obtaining 2a as a yellow syrup (28% yield, 190 mg). ^1^H NMR (500 MHz, CDCl_3_) δ ppm: 6.32 (s, 1H, Ar-H), 6.24 (s, 1H, Ar-H), 6.03 (br s, 1H, OH), 5.58 (br s, 1H, H-2), 4.53 (s, 1H, H-9), 4.41 (s, 1H, H-9´), 4.04 (br s, 1H, H-1), 3.87 (t, *J* = 6.7 Hz, 2 H, CH_2_O), 2.52–2.45 (m, 3 H, H, CH_2_Ph), 2.27–2.21 (m, 1H), 2.10 (br m, 1H), 1.85–1.72 (m, 7 H), 1.64–1.58 (m, 2 H), 1.49–1.43 (m, 2 H), 1.40–1.29 (m, 15 H), 0.93 − 0.90 (m, 6 H). ^13^C NMR (100 MHz, CDCl_3_) δ ppm: 157.65, 155.89, 147.31, 142.69, 139.44, 124.88, 115.23, 111.00, 109.46, 103.97, 68.37, 46.33, 36.15, 35.68, 31.99, 31.71, 30.99, 30.34, 29.58, 29.55, 29.39, 28.08, 26.27, 23.79, 22.81, 22.69, 19.52, 14.23, 14.18. HRMS *m/z*: [M + H]^+^ calculated for: C_29_H_47_O_2_ 427.3576, found 427.3583.

When the reaction was carried out by using K_2_CO_3_ (1.32 g, 9.54 mmol, 6 eq.) in acetone (120 mL, under inert atmosphere, and 1-bromooctane (0.83 mL, 4.77 mmol, 3 eq.), after silica gel column chromatography (100:0.01 hexane: EtOAc), compound 2b was obtained as colourless syrup (12% yield, 103 mg). ^1^H NMR (500 MHz, CDCl_3_) δ ppm: 6.28 (s, 2 H, Ar-H), 5.24 (s, 1H, H-2), 4.45 (s, 1H, H-9), 4.40 (s, 1H, H-9´), 4.03 (m, 1H, H-1), 3.89–3.81 (m, 4 H, 2 x CH_2_O), 2.96 (m, 1H, CH_2_Ph), 2.50 (t, *J* = 10.6 Hz, 2 H), 2.17–2.12 (m, 1H), 1.98 (d, *J* = 13.6 Hz, 1H), 1.76–1.70 (m, 6 H), 1.61–1.56 (m, 6 H), 1.46–1.43 (m, 4 H), 1.35–1.26 (m, 22 H), 0.91 − 0.88 (m, 9 H). ^13^C NMR (100 MHz, CDCl_3_) δ ppm: 149.76, 141.80, 130.74, 126.91 (2), 118.70, 109.73, 105.17, 45.03, 36.58, 36.36, 32.03, 31.88, 31.18, 30.99, 29.85, 29.83, 29.79, 29.62, 29.44, 26.37, 23.59, 22.83, 22.72, 19.48, 14.25, 14.23. HRMS *m/z*: [M + H]^+^ calculated for: C_37_H_63_O_2_ 539.4828, found 539.4839.

#### Reaction of CBD with amino acids. Synthesis of compound 4 (Scheme 4)

In a 100 mL round-bottom flask, CBD (500 mg, 1.59 mmol, 1 eq.), Et_3_N (0.89 mL, 6.36 mmol, 4 eq.), and CH_2_Cl_2_ (12 mL) were combined. Separately, in another 100 mL round-bottom flask, 4-nitrophenyl chloroformate (1.28 g, 6.36 mmol, 4 eq.) was dissolved in of CH_2_Cl_2_ (26 mL). Both solutions were cooled to 0 °C. Once the temperature was reached, the CBD solution was slowly added to the 4-nitrophenyl chloroformate solution, maintaining the temperature and stirring. The reaction mixture was stirred at room temperature for 15 min, then it was allowed to reach room temperature and stirred for an additional 1 h. After this period, CH_2_Cl_2_ was added. The dichloromethane solution was subsequently washed with 3 M HCl (3 × 10 mL), saturated brine (1 × 10 mL) and water (1 × 10 mL). The organic phase was then dried over anhydrous MgSO_4_, and finally, concentrated under reduced pressure. The residue was purified by column chromatography with silica gel (15% EtOAc in Hexane), yielding product **3** (82% yield, 842 mg), as a yellowish syrup. ^1^H NMR (500 MHz, CDCl_3_) δ ppm: 8.05 (d, *J* = 9.2 Hz, 4 H), 7.26 (d, *J* = 9.3 Hz, 4 H), 6.80 (s, 2 H), 5.07 (s, 1H), 4.36 (s, 1H), 4.32 (s, 1H), 3.62–3.58 (m, 1H), 2.57 (t, *J* = 12.9 Hz, 1H), 2.38 (t, *J* = 15.7 Hz, 2 H), 2.22–1.96 (m, 2 H), 1.82–1.78 (m, 1H), 1.65–1.50 (m, 3 H), 1.39–1.25 (m, 5 H), 1.09–1.03 (m, 5 H), 0.64 (t, *J* = 6.9 Hz, 3 H). ^13^C NMR (100 MHz, CDCl_3_) δ ppm: 155.33, 150.58, 149.48, 147.55, 145.43, 143.05, 134.77, 126.58, 125.34, 122.59, 121.40 (2), 111.40, 45.84, 38.31, 35.17, 31.30, 30.31, 30.26, 28.67, 23.66, 22.39, 19.62, 13.95. HRMS *m/z*: [M + Na]^+^ calculated for: C_35_H_36_N_2_O_10_Na 667.2268, found 667.2268.

A solution of compound 3 (570 mg, 0.88 mmol, 1 eq.) in anhydrous CH₂Cl₂ (50 mL) was prepared in a 100 mL round-bottom flask, under an inert atmosphere (N₂). *L*-valine methyl ester (592 mg, 3.54 mmol, 4 eq.) and DMAP (432 mg, 3.54 mmol, 4 eq.) were then added to this solution. The mixture was stirred under reflux for 12 h. Once the reaction was complete, monitored by TLC, the reaction mixture was filtered and concentrated under vacuum. Finally, the crude reaction product was purified by column chromatography with silica gel (22% EtOAc in hexane), yielding 4 (45% yield, 252 mg), as a colourless syrup. ^1^H NMR (500 MHz, CDCl_3_) δ ppm: 6.72 (s, 2 H), 5.45 (s, 2 H), 5.25 (s, 1H), 4.49 (s, 1H), 4.43 (s, 1H), 4.27 (q, *J* = 4.7 Hz, 2 H), 3.71 (s, 6 H), 3.59–3.57 (m, 1H), 2.56 (t, *J* = 11.1 Hz, 1H), 2.47 (t, *J* = 24.5 Hz, 2 H), 2.19–2.12 (m, 3 H), 1.98–1.94 (m, 2 H), 1.76–1.67 (m, 2 H), 1.58–1.50 (m, 6 H), 1.29–1.18 (m, 5 H), 0.94 (dd, *J* = 24.5, 7.0 Hz, 12 H), 0.82 (t, *J* = 7.0 Hz, 3 H). ^13^C NMR (100 MHz, CDCl_3_) δ ppm: 172.21, 154.03, 149.59, 147.66, 141.60, 132.08, 126.52, 124.32, 119.85, 111.00, 59.17, 52.12, 45.73, 37.99, 35.20, 31.44, 31.20, 30.40, 30.35, 28.91, 23.40, 22.37, 19.42, 18.84, 17.62, 13.93. HRMS *m/z*: [M + Na]^+^ calculated for: C_35_H_52_N_2_O_8_Na 651.3622, found 651.3616.

### Solubility

The solubilities of conjugates were determined using the shake flask method at 25 °C and 1 bar (Tavcar et al., 2024). Approximately, 20 mg of 1a,b, 2a,b or 4 were weighed into 5 mL plastic tubes. Solvents (water, *n*-hexane and isooctane) were added by volume in such quantities that conjugates were not dissolved entirely throughout the experiment, and a precipitate remained. The tubes were placed in an ultrasonic homogenizer (Bandelin Sonoplus) for 15 min. The samples were then centrifuged for 15 min at 5000 rpm and 25 °C. Each supernatant was filtered through a filter (0.22 μm nylon pore size syringe filter) and the solid weighed. It was dissolved in 3 mL ethanol and homogenized with the ultrasonic homogenizer for 5 min. Each solution was further diluted to achieve the concentration range corresponding to the linearity of the HPLC measurement curve and subjected to HPLC analysis (see “General Remarks” section). The highest measured concentration for each compound in each solvent was therefore used as the solubility value.

### Cell lines

Cell line used in this study was A549, an adenocarcinoma epithelial line of non-small cell lung cancer (NSCLC), from the Instrumentation Service Center of the University of Granada (Spain). Cells were growth in high glucose Dulbecco`s Modified Eagle`s Medium (DMEM) from Sigma-Aldrich, supplemented with 10% Fetal bovine serum (FMS) and 1% penicillin-streptomycin. The air condition was 37 °C at 5% of CO_2_.

### Cytotoxicity assay

A549 cells were grown at a density of 5 × 10^3^ per well in 48-well plates. After 24 h, cells were exposed to the compounds 1a, b, 2a, b and 4 at concentrations between 1 and 40 µM for 72 h in 37 °C and 5% of CO_2_ concentration. DMSO (0.08%) was used as solvent control. An MTT assay was used for the measurement of cytotoxicity, incubating the cells with MTT solution (5 mg MTT / mL PBS) at 10% well volume during 3 h until the formation of formazan crystals. After the incubation, crystals were dissolved with 8:1 solution of DMSO: Sorensen and the optical density (OD) was quantified with a spectrophotometer at 570 and 630 nm, subtracting the OD of the culture medium and MTT reagent without cells (blank). The percentage of relative inhibition was calculated with the formula:

$$\:\%\:Relative\:Inhibition=100-\left(\frac{\left(OD\:sample\:570-OD\:sample\:630\right)-blank}{\left(OD\:control\:570-OD\:control\:630\right)-blank}*100\right)$$

### Wound-healing assay

Wound-healing assay allows the quantification of the capacity of the drugs in the migration modulation. A549 cells were seeded in 12 well plates at a density of 3 × 10^5^ in 1 mL of completed DMEM and incubated 24 h until 100% confluence. After incubation, the wound was made profiling the well diameter with a 100 µL pipette tip, subsequently removing detached cells with a PBS wash and adding FBS-free DMEM. Cells were incubated in presence of the different compounds at IC_10_ concentrations for 72 h at 5% of CO_2_ and 37 °C. During the incubation, images were obtained at different times (0, 24, 48 and 72 h) with an inverted light microscope. The healing of the wound was analysed with the Wound Healing Tool extension of the free software ImageJ.

### Cell colony assay

Cells were seeded in 5 mL of complete DMEM at a density of 1 × 10^5^ cell in T25 falcon. After 24 h, compound treatments were added at IC_50_ concentration and incubated for 72 h at 5% of CO_2_ and 37 °C. After the incubation, the pre-treated cells were detached with a 1:2 solution of trypsin: PBS-EDTA, and the surviving cells were seeded at a density of 2 × 10^2^ cells per well in 12 well-plates in 1 mL of complete DMEM for 7 days at 37 °C and 5% of CO_2_. For the cell colony quantification, cells were stained with 0.08% sulforhodamine B solution (Sigma-Aldrich) in 1% glacial acetic acid (PanReac AppliChem). The percentage of colony formation have been represented with the formula:

$$\:\%\:Colony\:Formation=\frac{Number\:of\:colonies\:formed}{Number\:of\:cells\:seeded\:in\:a\:well}$$

### Statistical analysis

All the results were presented as mean ± standard deviation (SD) of triplicate cultures. Statistical analysis was performed using Student’s t-tests with the Statistical Package for the Social Sciences (SPSS) v.26 software. Data with *p* < 0.05 were considered as statistically significant.

## Results

### Synthesis

**Scheme 3:**
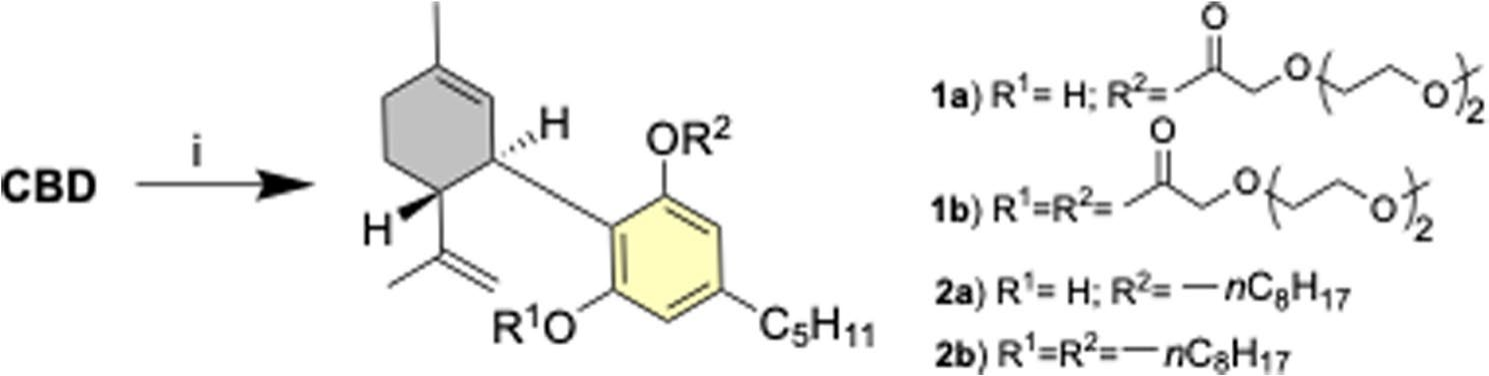
Synthesis CBD conjugates. For 1a,b, i) 2-[2-(2-methoxyethoxy)ethoxy]acetic acid (1.1 or 2.2 eq.), DCC, DMAP, CH_2_Cl_2_, 12 h, rt. For 2a,b, i) CBD, 1-bromooctane (1.5 or 3.0 eq.), K_2_CO_3_, acetone, 12 h, rt

Oligo(ethylene glycol) conjugates 1a,b have been prepared in good yields by esterification of CBD (Scheme 3). 2-[2-(2-Methoxyethoxy)ethoxy]acetic acid, used as the OEG chain block provider, was made to react with CBD under N_2_ atmosphere, using DCC as a coupling agent to activate the carboxylic acid, and DMAP as a basic catalyst.

During the process, 2-[2-(2-methoxyethoxy)ethoxy]acetic acid was used in different proportions to control the degree of CBD substitution. For the preparation of compound 1a, 1.1 eq. of acid were added, yielding only the monosubstitution product in a 67% yield. For the formation of compound 1b, the amount of acid was increased to 2.2 eq. to favour the disubstitution, resulting in the formation of compound 1b in a 60% yield.

For non-polar improved solubility of CBD, CBD–alkyl conjugates 2a,b were prepared by treating CBD with 1-bromooctane under an inert atmosphere of N_2_, using K_2_CO_3_ as a base and acetone as a solvent (Scheme 3). 1-Bromooctane was used in different proportions to control the degree of CBD substitution. In the optimized procedure, 1.5 eq. of the alkyl halide were used to obtain the monosubstitution product 2a (58% yield), while 3 eq. were added to promote the disubstitution, obtaining compound 2b (42% yield).

**Scheme 4:**
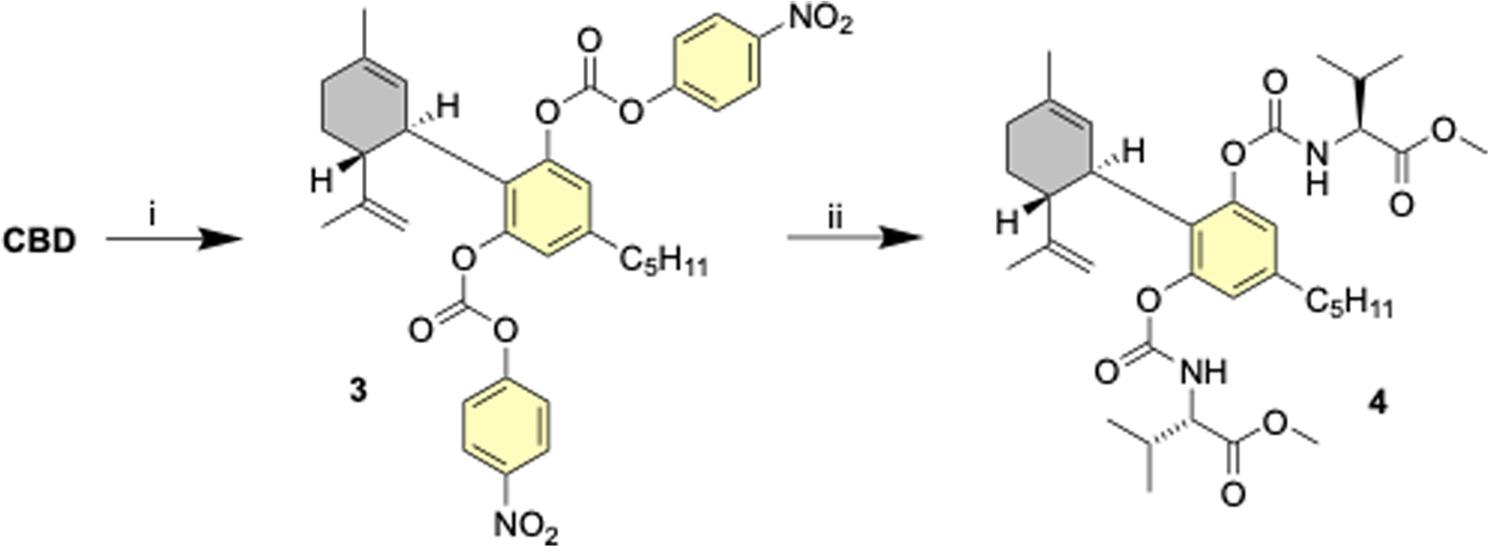
Synthesis of compound 4. (i) 4-Nitrophenyl chloroformate (4 eq.), Et_3_N, CH_2_Cl_2_, 1 h, rt; (ii) *L*-valine methyl ester (4 eq.), DMAP, CH_2_Cl_2_, 12 h, reflux

Amino acid conjugates of CBD were synthesized by treating CBD with 4-nitrophenyl chloroformate under an N_2_ atmosphere, using triethylamine as a base and CH_2_Cl_2_ as solvent, resulting in the formation of compound 3 in an (82% yield). Subsequently, 3 was reacted with *L*-valine methyl ester under an N_2_ atmosphere, using DMAP as a basic catalyst in CH_2_Cl_2_, resulting in the formation of 4 (45% yield) (Scheme 4).

### Solubility

We chose *oligo*(ethylene glycol) and alkyl substituents since it is known they improve solubility in very insoluble drugs (Fuentes-Ríos et al. 2022). Solubility tests of the conjugates 1a,b, 2a,b and 4 were carried out in water and glycerine, and in *n*-hexane and isooctane, as representative CBD-very low solubility polar and non-polar solvents, respectively. Results are shown in Table 1.

**Table 1:** Measured solubility of conjugates 1a,b, 2a,b, 4 and CBD.^(a−d)^

Nº	Product	Water	Glycerine	*n*-Hexane	Isooctane
*1*	**1a**	0.05 ± 0.02	0.09 ± 0.02	115.10 ± 0.07	22.1 ± 0.1
*2*	**1b**	0.17 ± 0.02	0.28 ± 0.03	130.11 ± 0.09	37.1 ± 0.1
*3*	**2a**	0.0 ± 0.02	0.02 ± 0.01	140.72 ± 0.10	55.8 ± 0.3
*4*	**2b**	0.01 ± 0.02	0.04 ± 0.01	154.21 ± 0.15	87.7 ± 0.5
*5*	**4**	0.10 ± 0.02	0.25 ± 0.05	171.41 ± 0.20	87.3 ± 0.7
*6*	**CBD**	0.012 ± 0.003 (0.0126)	0.02 ± 0.01 (0)	112.03 ± 0.02 (115.61)	32.10 ± 0.01 (28.37)

^(a)^ In parenthesis, CBD reported solubilities (Tavcar et al., 2024). ^(b)^ Solubilization essays were carried out as detailed in Experimental. ^(c)^ Units in mg/mL at 25 °C. ^(d)^ Results are expressed as the mean ± SD and the experiment was made in triplicate measurements.

### Antitumoral assays

**Fig. 1:**
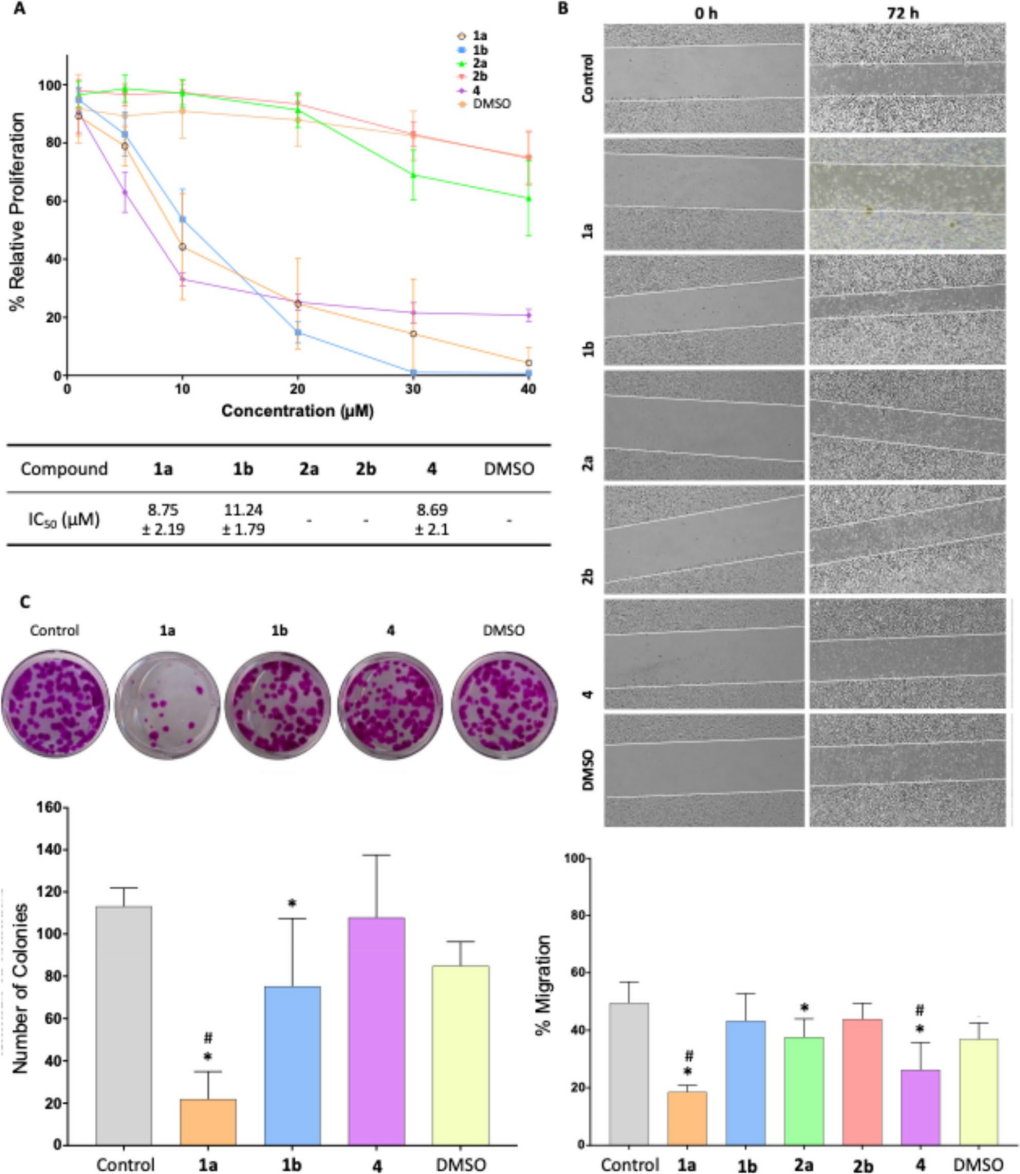
Graphic representation of the relative proliferation (%RP) of the compounds 1a,b, 2a,b and 4 in concentrations of 1–40 µM at 72 h exposure (**A**). Image representation of cell migration after 72 h treatment with IC_10_ concentrations of the compounds 2, 3, 4, 5, and 7 and its graphical representation (**B**). Graphical representation of the number of colonies after 72 h pre-treatment with IC_50_ concentrations of compounds 1a, 1b and 4 (**C**). Asterisk “*” represents a significant difference between the treatment and the control with a *p* > 0.05. Hash “#” represents a significant difference between the treatment and DMSO

The percentage of relative proliferation (%RP) at increasing doses of the different samples was represented in Fig. 1.A. Among all the CBD analogues tested, only compounds 2a and 2b have not reached an IC_50_ value under 40 µM. On the other side, compounds 1a, 1b and 4 reached the IC_50_ values of 8.75 ± 2.19, 11.24 ± 1.79 and 8.69 ± 2.1 µM, respectively. Solvent-only treatment with DMSO did not affect the A549 cells proliferation, being the cytotoxic effect of the compound treatments due to the drugs effect.

As shown in Fig. 1.B, compounds 1a, 1b and 4 had an inhibitory effect in the cell migration after 72 h of exposure. DMSO dose also reduced the A549 cell migration in comparison with control cells. Among the compounds with antimigratory effect, only the compound 1b had higher and significant effect in comparison with the solvent-only treatment with a 18.53% of migration, 66.7% lower in comparison with control treatment. On the other hand, the colony formation assay allows to study the proliferation capacity of pre-treated A549 cells and its ability to settle in a new tissue and generate a new tumour after the migration. This assay requires a pre-treatment concentration of IC_50_, so only the compounds that reached that value among the 1–40 µM (compounds 1a, 1b and 4) were tested. This colony formation after the treatment of the CBD analogues 1a, 1b and 4 was represented in Fig. 1.C. Only the compound 1b inhibits the capacity of proliferation of pre-treated cells, reducing the colony formation by 80% in comparison with control treatment.

## Discussion

### Synthesis

**Fig. 2:**
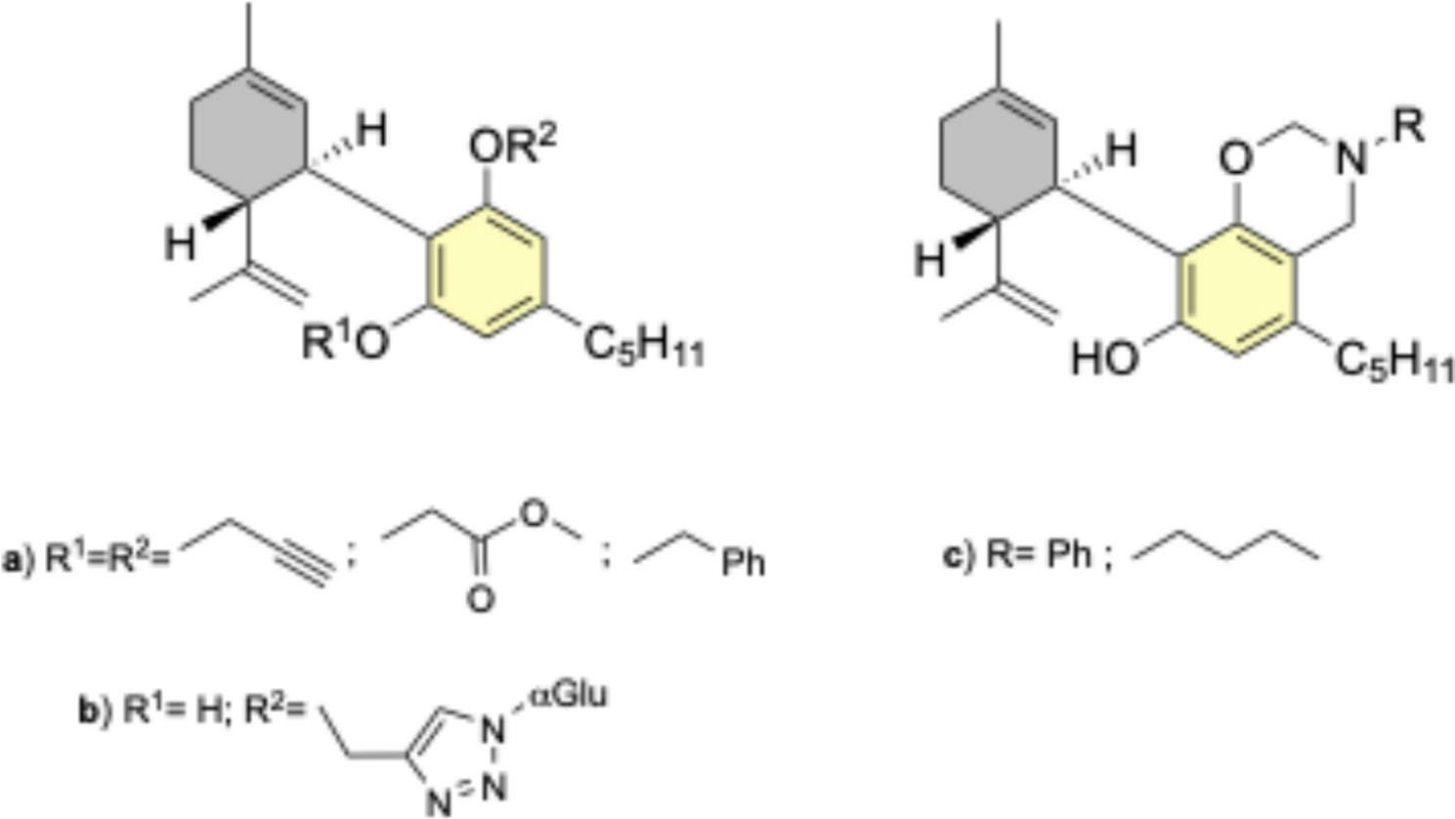
Reported conjugates of CBD prepared by structural modification of the cannabinoid. **a**) Alkylation of hydroxyl groups: (i) HO-CH_2_-C ≡ CH, Ph_3_P, DIAD, THF; (ii) Br-CH_2_-COOCH_3_, K_2_CO_3_, DMF; (iii) Br-CH_2_-Ph, K_2_CO_3_, DMF. **b**) Monosubtitutions: (i) Br-CH_2_-C ≡ CH, K_2_CO_3_, DMF; (ii) α-azidoglucose, copper (II) acetate, sodium ascorbate, *t*BuOH-H_2_O (v: v, 1:1), THF, 2 h, rt. **c**) Annular conjugates: (i) Ph-NH_2_, HCHO, MeOH. (ii) CH_3_-CH_2_-CH_2_-CH_2_-NH_2_, HCHO, MeOH

There are not many examples of previously prepared *O*-alkylated CBD conjugates. For example, alkynyl, ester and benzyl derivatives have been prepared by substitution of both hydroxyl groups present in CBD (Fig. 2.a), in a comparison between the classical alkylation of phenolic alcohols with alkyl halides and a protocol based on the Mitsunobu reaction, in which the CBD hydroxyls act as the acid component (Ziegler et al., 2021).

Hydroxyl monosubstitution has also been achieved by the synthesis of a glycosylated CBD conjugate (Cheng-Ting et al. 2022). Derivatization was achieved through the hydroxyl group by alkynylation followed by reaction with an azide derivative of glucose, in a classical Click reaction. Glucosyl derivative was prepared to improve the water solubility of the cannabinoid and to evaluate the cytotoxicity and anti-inflammatory properties of new conjugate (Fig. 2.b) (Cheng-Ting et al. 2022). Moreover, CBD conjugates including an oxazinane fused ring have also been prepared with the aim of evaluating the biological activity of new derivatives (Fig. 2.c). Annulation is carried out through the hydroxyl groups by following the Mannich procedure, leading the formation of substituted oxazinyl rings (Pankaj-Singh et al. 2024).

In the present study, we report an improvement in CBD solubility through the synthesis of labile conjugates, which were designed to release CBD under physiological conditions: ester derivatives can be easily hydrolysed under slightly acid or basic conditions, but also by the hydrolase action, releasing the active molecule, namely acting as a prodrug.

CBD–Oligo(ethylene glycol) conjugates, compounds 1a,b, have been prepared by esterification of CBD (Scheme 3) (Narsimha et al. 2012). The presence of the ethylene glycol moieties will directly contribute to a CBD–improved solubility. Modulating the 2-[2-(2-methoxyethoxy)ethoxy]acetic acid proportion to CBD we were able to control the degree of CBD substitution, separately synthesizing in good yields 1a and 1b. The reaction of CBD with 2.2 eq. of acid afforded 1b, and only a small amount of 1a (2%). ^1^H NMR integrals confirmed the substitution pattern. For compound 1a, in the 7.0 to 6.0 ppm range, three singlets are present, each one corresponding to one proton, which can be assigned to the two aromatic protons, as well as the proton of the hydroxyl group (Figure SI1). Therefore, this pattern is consistent with the structure of the monosubstituted product. However, in the same range, for compound 1b, only a single singlet of two protons integral is observed (Figure SI3). This is consistent with the two equivalent aromatic protons in the disubstituted product 1b.

We also accomplished the preparation of lipid soluble derivatives (Scheme 3). Alkyl conjugates 2a,b have been obtained by treating CBD with 1-bromooctane, since it contains a long aliphatic chain in its structure, which can contribute to improved solubility through the formation of micellar systems. As above, modulation of the 1-bromooctane content during the reaction allowing to control the degree of CBD substitution, separately affording monosubstitution and disubstitution products 2a and 2b, respectively. Selectivity of the reaction was confirmed by ^1^H NMR: only one 2 H-integral singlet is observed at 6.28 ppm for aromatic protons of 2b, while two singlets, 1H-integral each, are observed at 6.32 and 6.24 ppm for 1b (Figure SI7 and SI5, respectively).

To carry out a comparative analysis of activity and solubility, we prepared amino acid conjugates of CBD by activating hydroxyl groups of CBD with 4-nitrophenyl chloroformate (Reddy-Yerramreddy et al. 2010), followed by treatment with *L*-valine methyl ester as representative amino acid (Scheme 4). The presence of amino acids will also improve solubility in hydrophilic solvents. As mentioned, the synthesis of compound 4 was carried out in two stages. First, activation of hydroxyl groups with 4-nitrophenyl chloroformate obtaining 3: this derivative served as a precursor for compound 4, as it contains two activated carbonate groups susceptible to nucleophilic attack. Second, reaction with *L*-valine methyl ester, resulting in the formation of 4 (Scheme 4). Unlike the previous syntheses, this time the objective was to obtain exclusively the disubstitution product of both compounds 3 and 4. To achieve this, instead of adjusting the proportions of 4-nitrophenyl chloroformate and *L*-valine methyl ester, the reactions were carried out in excess, using 4 eq. of reagent in both cases. NMR data confirmed the substitution pattern (Figures SI9-SI12).

### Solubility

Solubility in water and hydrophilic solvents is a major issue in drug design (Kim et al. 2021), since it is necessary for biological applications of active compounds. This is a major issue for CBD, because the terpenoid skeleton of CBD has a markedly reduce solubility in water and polar solvents, as mentioned above, 12.6 mg/L in water (inherently insoluble). It is hydrophobic, meaning it floats on water rather than dissolving, which causes poor oral bioavailability (approx. 6%). To make it miscible for food or pharmaceutical applications, emulsions or nanocarriers must be used. These systems include polymeric micelles and nanoparticles (such as PLGA and zein/whey protein), hybrid nanoparticles jelled in cross-linked chitosan, polymeric drugs and lipid formulations like nanostructured lipid carriers, vesicles, or nano- and microemulsions (Grifoni et al. 2022; Fuentes-Ríos et al. 2024). It has also been shown that polymeric *p*-vinyl pyridine and *p*-isopropylacrylamide nanoparticles allowed to increase the CBD solubility by more than 60-fold (Moya-Utrera et al. 2024).

Due to its therapeutical interest, solubility of CBD has recently been studied both from an experimental and theoretical point of view (Pulido-Bonilla et al. 2023; Tavcar et al., 2024). Remarkably, CBD solubility in isooctane has been reported as one of the smaller one in non-polar solvents (28.37 mg/mL), while in water 0.0 mg/mL has also been reported (Table 1, entry 6) (Tavcar et al., 2024).

As can be seen in Table 1, all compounds present a qualitatively superior solubility to that of CBD measured at 25 °C. As expected, OEG conjugates 1a,b are the most soluble in water and glycerine: 1a reaches 0.05 mg/mL in water and 0.09 mg/mL in glycerine, while 1b shows solubilities of 0.17 and 0.28 mg/mL in each solvent, respectively. These results indicate a 14-fold enhancement in the aqueous solubility of 1b relative to CBD, which requires about 86 mL of water to dissolve an equivalent amount of material. In this sense, it is important to remark that the presence of the OEG chains in 1a, b increase the water solubility, maintaining the solubility in apolar solvents. The last is good and similar to that of CBD, with values of 115.10 mg/L for 1a and 130.11 for 1b in *n*-hexane, and 22.1 for 1a and 37.1 for 1b in isooctane (Table 1, entries 1 and 2). On the other hand, compounds 2a, b, having one and two alkyl chains, respectively, in the structure, do not substantially improve the solubility in hydrophilic solvents (Table 1, entries 3 and 4). Remarkably, conjugate 4, bearing two *L*-valine moieties and thus incorporating two amide functionalities, exhibits a substantial improvement in CBD solubility across all tested solvents: 8-fold in water, 13-folds in glycerine, 1.5-folds in *n*-hexane and nearly 3-folds in isooctane (Table 1, entry 5).

### Antitumoral activity

Regarding the antitumour activity of CBD, our previous study shows an IC_50_ value of 15.99 ± 0.41 with the non-conjugated CBD treatment, so the compounds 1a, 1b and 4 not only had an increased solubility in comparison with the CBD but also improve the cytotoxic activity against A549 cells (Fuentes-Ríos et al. 2024). There are some examples of the anti-tumoral potential of CBD analogues. For example, the quinone derivate CBD hydroquinone maintain an antitumoral effect in 2D and 3D colonies of colorectal SW-620 cells, but its cytotoxic effect is lower than the original CBD (Beben et al. 2024). Some CBD piperazinyl derivates shown an increased antitumoral effect, reducing IC_50_ concentration up to 5.5 times in comparison with the original molecule (Chen et al. 2024). Beside the proliferation capacity, another hallmark of cancer is the ability to migrate to other areas of the body, allowing tissue invasion and producing metastasis (Esteller et al. 2024).

Likewise, the inhibitory effect of CBD on A549 cell migration has been previously described in vivo and in vitro (Ramer et al. 2011; Milian et al. 2020), with an anti-migratory capacity similar to that of compound 1b (Fuentes-Ríos et al. 2024). This migratory inhibition could be related to interaction with some A549 TRPV1, CB1, and CB2 membrane receptors, as has been demonstrated with some cannabinoids such as CBD (Yan et al. 2023; Seltzer et al., 2020; Ramer et al. 2010).

Regarding colony formation, we have previously demonstrated that unconjugated CBD can reduce the proliferation of pretreated cells by 100% (Fuentes-Ríos et al. 2024). However, among the conjugated CBDs, only compound **1a** showed an 80% decrease. This reduction in the activity of the CBD molecule after conjugation could be related to a difficulty in interacting with cannabinoid receptors (Milian et al. 2020).

## Conclusion

CBD conjugates 1a, b, 2a, b, 3 and 4 were successfully prepared in good yields through selective esterification reaction of the phenolic hydroxyl groups. The degree and nature of substitution were controlled using 2-[2-(2-methoxyethoxy)ethoxy]acetic acid or 1-bromooctane in different proportions. Activation of the phenolic hydroxyl groups with 4-nitrophenyl chloroformate, followed by *L*-valine methyl ester treatment, enabled efficient incorporation of amino acid moieties into CBD scaffold.

Among the synthesized derivatives, the *oligo*(ethylene glycol) conjugates 1a, b were the most soluble in water and glycerine, with compound 1b showing up to a 14-fold increase in water solubility relative to CBD. In contrast, alkyl substituted conjugates, 2a, b, provided only slightly improvements in solubility in non-polar solvents. Remarkably, conjugate 4, bearing two L-valine moieties in the structure, demonstrated a substantial enhancement of solubility across all the tested solvents, achieving up to a 13-fold increase in glycerine. The incorporation of OEG chains in 1a, b significantly increases their water solubility, maintaining the solubility in apolar solvents.

Biological evaluation revealed that compounds 1a, 1b and 4 exert enhanced cytotoxicity against A549 lung adenocarcinoma cells (IC_50_ ranging from 8.69 to 11.24 µM), compared to native CBD (IC_50_ = 15.99 µM). Additionally, 1b demonstrated the most pronounced antimigratory and colony formation inhibitory effects reducing cell migration.

Overall, the improved solubility profiles of these CBD conjugates, particularly in water and polar solvents, represent a significant advancement over the parent compound. These findings support the potential of such semisynthetic CBD derivatives as promising candidates for further pharmacological development. The good solubility in water and polar solvents of conjugates, remarkably improving that of CBD, makes them potential useful semisynthetic drugs.

## Supplementary Information

Supplementary Material 1: NMR spectra of conjugates.

## Data Availability

This manuscript does not report data generation or analysis.
