# Supplementary material for: Solubility-improved and antitumor activity of (-)-cannabidiol conjugates
Source: J Cannabis Res. 2026 May 28;8:91. doi: 10.1186/s42238-026-00453-5 (PMC13450066; doi:10.1186/s42238-026-00453-5)
Supplement: Supplementary file 1 [file 42238_2026_453_MOESM1_ESM.docx]

**SUPPLEMENTARY INFORMATION**

**Solubility-Improved and Antitumor Activity of (-)-Cannabidiol Conjugates**

Daniel Crespo,^a,b^ Javier Moreno, ^c,d,e^ Cristina Mesas,^c,d,e^ Amelia Díaz,^a,b^ Iván Cheng-Sánchez, ^a,b^ Francisco Sarabia,^a,b^ J. Manuel López-Romero,^a,b*^ Consolación Melguizo,^c,d,e^ José Prados^c,d,e^

^a^	Dpto. Química Orgánica, Facultad de Ciencias, Universdad de Málaga, Boulevard Louis Pasteur 31, 29071 Málaga, Spain

^b^	Instituto Universitario de Materiales y Nanotecnología, IMANA, University of Málaga, Campus de Teatinos, 29071, Málaga, Spain

^c^	Instituto de Investigación Biosanitaria de Granada (ibs. GRANADA), 18014 Granada, Spain

^d^	Institute of Biopathology and Regenerative Medicine (IBIMER), Center of Biomedical Research (CIBM), University of Granada, 18100 Granada, Spain

^e^	Dep. of Anatomy and Embryology, Faculty of Medicine, University of Granada. 18071 Granada, Spain

* Corresponding author: J. M. López-Romero (jmromero@uma.es)

**Figure SI1.** ^1^H NMR of compound **1a** (500 Hz, CDCl_3_).

**Figure SI2.** ^13^C NMR of compound **1a** (100 Hz, CDCl_3_).

**Figure SI3.** ^1^H NMR of compound **1b** (500 Hz, CDCl_3_).

**Figure SI4.** ^13^C NMR of compound **1b** (100 Hz, CDCl_3_).

**Figure SI5.** ^1^H NMR of compound **2a** (500 Hz, CDCl_3_).

 **Figure SI6.** ^13^C NMR of compound **2a** (100 Hz, CDCl_3_).

**Figure SI7.** ^1^H NMR of compound **2b** (500 Hz, CDCl_3_).

**Figure SI8.** ^13^C NMR of compound **2b** (100 Hz, CDCl_3_).

**Figure SI9.** ^1^H NMR of compound **3** (500 Hz, CDCl_3_).

**Figure SI10.** ^13^C NMR of compound **3** (100 Hz, CDCl_3_).

**Figure SI11.** ^1^H NMR of compound **4** (500 Hz, CDCl_3_).

**Figure SI12.** ^13^C NMR of compound **4** (100 Hz, CDCl_3_).
